# Editorial: Modern neurosurgical management of gliomas, including local therapies

**DOI:** 10.3389/fonc.2023.1217180

**Published:** 2023-08-08

**Authors:** Aldo Spallone

**Affiliations:** Institute of Bioorganic Chemistry (IBKh) Institute, Russian Academy of Sciences (RAS), Moscow, Russia

**Keywords:** glioblastoma, surgery, chemo-radio therapy, BNCT (boron neutron capture therapy), microenvironment resistance

Glioblastoma (GBM) remains one of the most difficult to treat diseases in the field of oncology, and in neurooncology in particular. This Editorial focuses on the role of surgery and includes the four manuscripts included in the Research Topic “*Modern Neurosurgical Management of Gliomas, including Local Therapies*”.

of Despite extensive research and significant improvement in operative techniques and diagnostic technology, patient survival has barely increased in the last 40 years: from slightly more than one year to less than two years (1, 2). Moreover, this improvement was achieved by using some very aggressive management protocols, including repeated surgery performed in on an awake patient, as in many surgical experiences, including my own (3, 4). Currently, surgery represents a major instrument in the hands of clinicians in for helping these unfortunate patients. In fact, it is well known that apparent radical surgery is a key factor for prolonging post-operative patients’ survival [(5, 6), Ren et al. (7, 8)], in as well as reoperative surgery patients (3, 9).

This the necessitates of searching for any possible means in to increase the chances of achieving an apparent surgical radical excision in demanding cases, such as tumors located in critical areas and/or deeply situated in the brain, as well as in recurrent tumors. In this respect, the contribution of Ren et al. is of relevant importance.

In fact, using the sophisticated method the authors described, with the patient either under anesthesia or awake, the surgeon can be very aggressive in removing the tumor, while in at the same time, remaining very safe as regards the risk of postoperative loss of functions, in particular motor functions. Moreover, monitoring the minimal subcortical monopolar threshold (MSCMT) can accurately predict the actual risk of damaging the pyramidal tracts; therefore, this unfortunate occurrence can be avoided. The authors actually define a clear cut-off that can be used not only for preventing post-operative deficits, but also for predicting progression-free survival and prognosis of operated GBM patients.

Prognostic indicators for GBM patients can also be retrieved from the experience of other surgical specialties, demonstrated by Li et al., who adapted a protocol originally implemented in Italy for abdominal cancer to malignant glioma patients in for (10). These authors transferred a concept to neuro-oncolological patients, introduced for colorectal cancer by an Italian group, and found that the inflammatory and nutritional status influenced the overall survival of GBM patients. Indeed, serum albumin, concentrate cholesterol level, and the neutrophils to lymphocytes ratio were significantly associated with OS in their patients, and were shown to be reliable prognostic indicators. Wang et al. reported the excellent results of for the implementation of an enhanced post-operative recovery protocol in the early stage of glioblastoma- operated patients. In this case, the authors also adapted protocols already reported by other surgical specialties to neuro-oncology patients (11). In fact, in the last decade, great attention has been paid in different surgical fields to the positive impact of early post-operative recovery in oncological patients. GBM patients are not an exception, and the study of Wang at al. gives another convincing demonstration of this concept.

Zerdan et al. provided a very thoughtful and complex analysis of the recent relevant literature on glioma biomarkers, with a clear view on the implementation of potential future therapeutic protocols. This review is extremely comprehensive. Data are provided accurately and concisely, with the relevant message that future, hopefully effective treatment of gliomas, in particular of malignant ones, would require extensive and deep knowledge of these identified biomarkers.

Classical chemotherapy has seen the introduction of Temozolomide (TMZ) for improving survival in GBM, particularly in patients possessing promoter methylation of the enzyme 0-6-methylguanine DNA methy|transferase (MGMT) [Zerdan et al. (12–14)]. Other, more aggressive therapeutic protocols employing combinations of different agents can be used as a salvage therapy in recurrent GBMs; however, their benefit as a routinely adopted therapeutic regime for these tumors is limited, mostly due to their potential toxicity (1).

Radiation therapy also has shown its efficacy if used as a whole brain radiation technique; and its efficacy is dose related. This indicates that the appropriate radiation dosage should be chosen according to the principle of balancing the positive effect of inducing tumor cell death against the risk of causing severe radionecrosis sequelae in the normal brain adjacent to the lesion (1, 12).

Positive results have been recently reported by a group including the author of this Editorial (9), using a system locally implemented into the surgical cavity at the end of the tumor removal procedure, with the aim of delivering a precisely calculated high radiation dosage targeted mostly at the edge of the surgical cavity wall where presumably scattered tumor cells are left *in situ* despite apparent macroscopic tumor removal. In this study, the survival of GBM patients treated with such a protocol was significantly longer than that of patients undergoing routine post-operative chemoradiotherapy, while in at the same time, radionecrosis sequelae were limited. However, the study was at single centre and retrospective; and certainly these interesting results await confirmation.

Boron capture radiation therapy (BRT) has been given recent attention after being introduced with little success in clinical practice a few decades ago (15, 16). Although promising results have been reported recently by Japanese groups (16, 17), its real efficacy in the treatment of GBMs is still unproven and highly debated (18–21), and the ideal pharmacological agent to be used for better targeting of tumor cells by activated neutron particles remains to be identified (22). In this respect, very recent experimental studies have shown interesting results; again, they await clinical confirmation (23).

A promising way for to treat GBMs would be to better address immunotherapeutic protocols, which are likely to suffer from the shortcomings of having to fight against a very effective GBM microenvironment barrier, which does not allow immunocompetent cells, in particular T lymphocytes, to reach the tumor in such a way to be able to exert their anti-tumoral activity (24). In fact, the biology of immuno-related cells infiltrating GBM is still poorly understood and underlying partially clarified complex biological mechanisms can well explain why T lymphocytes, representing a non-negligible fraction of those cells which are found in the microenvironment of GBM, do not exert the antitumoral activity they exert in other types of cancer (25).

The definition of what constitutes the GBM microenvironment, as related to its capacity of making the tumor an immuno-resistant biological entity, is extremely complex. Extensive recent research in the field of neurogenetics has focused on the potential role of several biological factors in such a process (26–28). In particular, recent studies have convincingly demonstrated that CD4, CD8, and II-10 can play a major role in the development of an efficacious anti-immunity barrier, as in the case of GBMs (24, 27).

It is well known that myeloid-derived cells are the most prominent immunocompetent cells found in GBM (29–31), and they can have a direct immunosuppressive effect (24, 27). This role could be mediated by the regulatory B cells called Breggs, which exert an immunosuppressive effect controlled by the same GBM microenvironment (27). Perhaps an increased focus on the role of the B regulatory lymphocytes in GBM could be a clue for more effective immunologically based therapeutic protocols for these tumors.

of the Breggs can act in a more complex manner, which could include significant roles exerted by other bio factors such as CD 155, PD-1, and TGFbeta (27), in addition to the above mentioned CD4, CD8, and II-10. All this strongly suggests a crucial role of B lymphocytes in the development of the immunoresistance of GBM and future research should look in this direction. This effect would appear to be mainly mediated by II-10 via *the* JAK-STAT pathway (24, 32); and its selective inhibition seems to produce a positive effect in the clinical settings (24, 33), although this interesting observation awaits future confirmation.

Genetically based insights continue to be explored for GBM [(2), Zerdan et al. (34, 35)] in a personalized way, as suggested a few years ago (36). This likely represents the near future of GBM research focused on the issue of finding an effective treatment. The wide availability of excellent experimental GBM models (37) can certainly be relevant for exploring this potentially very interesting new direction.

## Author contributions

The author confirms being the sole contributor of this work and has approved it for publication.
